# Editorial: Rising stars in non-neuronal cells 2022

**DOI:** 10.3389/fncel.2024.1375949

**Published:** 2024-02-13

**Authors:** Camila Cabral Portugal, Maria Jose Bellini, Yu-Feng Wang

**Affiliations:** ^1^Instituto de Investigação e Inovação em Saúde, Universidade do Porto, Porto, Portugal; ^2^National Scientific and Technical Research Council (CONICET), Buenos Aires, Argentina; ^3^Zhejiang Chinese Medical University, Hangzhou, China; ^4^Harbin Medical University, Harbin, China

**Keywords:** microglia, tissue-resident macrophages, organoids, induced pluripotent stem cells (iPSC), cerebrovascular disease, choroid plexus, transient ischemic stroke, TGF-β3

The intricacies of the central nervous system (CNS) are profoundly shaped by non-neuronal cells (NNCs), whose roles extend far beyond mere support to actively dictating neural function and integrity. NNCs encompass astrocytes, oligodendrocytes, tissue-resident macrophages (TRM), including microglia and border-associated macrophages (comprising macrophages from the boundaries of the CNS, including meninges, vasculature, and the choroid plexus), ependymal cells, endothelial cells, pericytes, among other cell types. The biology and mechanisms underlying NNC activity remain poorly explored. This Research Topic highlights groundbreaking research and thoughts spearheaded by emerging leaders in the NNC domain, focusing on the enhancement or development of new techniques for studying microglia and cerebrovascular development in health and disease, highlighting the importance and involvement of the emerging leaders in the NNC domain to develop reliable approaches to study the NNCs.

Microglia are at the forefront of maintaining CNS homeostasis, known for their adaptability to environmental cues and critical roles in the CNS's immune responses and tissue-specific functions. The opinion article published by Cerneckis and Shi discusses the crucial roles of TRMs, including microglia and border-associated macrophages (BAMs), in CNS homeostasis and disease. It points out the limitations of existing human cell-based models in accurately replicating brain macrophage subtypes and molecular mechanisms. The article suggests using induced pluripotent stem cells (iPSC)-derived neuroimmune organoids to establish diverse human brain TRM models for studying their roles in tissue homeostasis and diseases like Alzheimer's. The study also discusses species-specific differences between human and murine microglia, emphasizing the need for robust human cell-based models to better understand human TRM biology. It outlines methods for differentiating macrophage and microglia-like cells from iPSCs and their potential applications in understanding brain macrophage diversity and functions. It marks a significant step toward understanding the complex roles of brain macrophages in health and disease using advanced human cell-based models.

Also, in pursuit of developing better models for studying microglia, Timmerman et al. using microglia from adult rhesus macaques (*Macaca mulatta*), addressed the gene expression disparities between *ex vivo* (freshly isolated) and *in vitro* (cultured) microglial samples. Utilizing *in-silico* (computational) and *in vitro* methods, this original research article investigates cues involved in the induction or maintenance of the *ex vivo* microglia reference transcriptome. Timmerman et al. used NicheNet, an *in-silico* tool, to explore CNS-derived cues that could explain differences between the transcriptomes of *ex vivo* and *in vitro* microglia. They identified High Mobility Group Box 2 (HMGB2) and interleukin (IL)-1β-associated signaling pathways as potential drivers of gene expression in cultured microglia.

This study also exposed primary microglia to conditioned media (CM) from neurospheres (composed of microglia, oligodendrocytes, and radial glia) or CM from purified oligodendrocytes at different developmental stages to observe their effects on microglia signature gene expression. By instructing NicheNet with these experiences and other data from the literature, they found that transforming growth factor beta 3 (TGF-β3) and laminin subunit alpha 2 (LAMA2) could modulate microglial signature gene expression levels. To test this hypothesis, authors cultivated microglia on laminin-coated substrates or exposed them to TGF-β3 and observed an increase in the mRNA levels of the microglia signature genes TREM2, GPR34, and P2RY13, along with a decrease in mRNA levels of extracellular matrix-associated genes MMP3 and MMP7 (Timmerman et al.). In order to preserve the microglial identity in culture by maintaining the expression of microglial signature genes, the authors suggest cultivating microglia in the presence of TGF-β3 on laminin-coated substrates to improve current microglial culture protocols (Timmerman et al.).

In the realm of cerebrovascular development, Travasso and Coelho-Santos delved into the intricate processes shaping the development of the brain's vasculature. The cerebrovascular system, encompassing arteries, capillaries, and veins, plays a crucial role in metabolic homeostasis by aligning the brain's heightened energy demands with the supply of nutrients and oxygen. This intricate orchestration forms a dynamic 3D network sculpted through biological responses, including angiogenesis, remodeling, and pruning of vascular endothelial cells (Travasso and Coelho-Santos).

This opinion article discusses the challenges in understanding cerebrovascular network development due to its complexity and poor accessibility during development. The need to use cutting-edge techniques like multiphoton microscopy for detailed, longitudinal study of the capillary network construction and mathematical modeling as a tool to analyze cerebrovascular network development, including blood flow and irrigation, is proposed to provide insight into the mechanisms of vascular adaptation *in vivo*, contributing to the understanding of cerebrovascular growth and maturation (Travasso and Coelho-Santos). In this insightful piece, Travasso and Coelho-Santos introduce the concept of image-based angio-adaptation modeling, presenting a potential breakthrough in understanding how vascular dysfunction and malformation contribute to neurodevelopmental and neurological diseases. This innovative approach holds promise for enhancing disease diagnosis, prognosis, and treatment, contributing to advancing personalized medicine in the near future.

The original article presented by Chen et al. has shown that transient ischemic stroke (TIS) triggers sustained damage of the choroid plexus blood-cerebrospinal fluid (CSF) barrier, inducing the recruitment of immune cells, thereby sustaining an immune-oriented microenvironment in the brain that contributes to neuroinflammation in the TIS subacute phase. Importantly, this original research article delineates several molecular mechanisms linked to this damage, including a temporary loss of the Ste20-related proline-alanine-rich kinase (SPAK) protein complex, an increase in SPAK-NKCC (Na^+^-K^+^-2Cl^−^-cotransporter 1) phosphorylation, and elevated proinflammatory lipocalin-2 mRNA and protein levels, unveiling new targets for brain damage treatments.

It is essential to recognize the multitude of critical questions in NNC research that are yet to be addressed. These include, but are not limited to, the development of optimal *in vitro* models that closely replicate *ex vivo* NNC data, the exploration of NNCs' influence on the CNS's structural and functional network, understanding of neuronal network dynamics, synaptic function, behaviors, energy metabolism, and the neurovascular unit, and how to overcome the heterogeneity of NNCs under different physiological and pathological conditions. This Research Topic addresses some of these questions by the internationally recognized researchers in the early stages of their careers, which is fundamental to safeguarding tomorrow's driving force in the innovation of studying NNCs and the CNS.

## Author contributions

CCP: Conceptualization, Writing—original draft, Writing—review & editing. MB: Writing—original draft, Writing—review & editing. Y-FW: Conceptualization, Writing—original draft, Writing—review & editing.

